# John Cunningham Virus and Progressive Multifocal Leukoencephalopathy: A Falsely Played Diagnosis

**DOI:** 10.3390/diseases12050100

**Published:** 2024-05-13

**Authors:** Dimitra S. Mouliou

**Affiliations:** Independent Researcher, 38500 Volos, Greece; demymoole@gmail.com

**Keywords:** JCPyV, PML, progressive multifocal leukoencephalopathy, multiple sclerosis, MS, HIV, AIDS, immunocompromised, false-positive, false-negative

## Abstract

Progressive Multifocal Leukoencephalopathy (PML) is a possibly fatal demyelinating disease and John Cunningham Polyomavirus (JCPyV) is believed to cause this condition. The so-called JCPyV was initially reported in lymphoma and Human Immunodeficiency Virus (HIV) cases, whereas nowadays, its incidence is increasing in Multiple Sclerosis (MS) cases treated with natalizumab (Tysabri). However, there are conflicting literature data on its pathology and diagnosis, whereas some misdiagnosed reports exist, giving rise to further questions towards the topic. In reality, the so-called PML and the supposed JCPyV are not what they seem to be. In addition, novel and more frequent PML-like conditions may be reported, especially after the Coronavirus Disease 2019 (COVID-19) pandemic.

## 1. Introduction

Progressive Multifocal Leukoencephalopathy (PML) was initially reported in 1958, in three cases with underlying cancer, out of whom two were chronic lymphatic leukemia patients and the other one was a man with Hodgkin’s disease [1]. Afterwards, John Cunningham Polyomavirus (JCPyV) was suspected in case of Hodgkin’s disease that presented PML, in 1971 [2].

PML is believed to be an opportunistic severe and often fatal demyelinating disease of the Central Nervous System (CNS), and JCPyV seems to be its causative agent [3]. JCPyV infection is evident globally, and its prevalence varies across ages, with older people showing higher incidence rates [4,5]. Although JCPyV establishes a persistent asymptomatic infection, immunosuppression in certain cases may set apart the subclinical phenotype [6]. Since nowadays immunomodulation is increasingly preferable, the general PML risk is rising [7]. Heretofore, there is evidence supporting JCPyV reactivation particularly in individuals with immunodeficiency syndromes or therapy-induced immunosuppression, and also in transplant recipients, autoimmune, hematologic and oncologic cases [8].

Except from the clinical manifestations and radiologic data, the definite PML diagnosis requires a neuropathological demonstration of the typical histopathological triad, in parallel with JCPyV identification [9]. Moreover, Polymerase Chain Reaction (PCR) assays and anti-JC serologic methods are widely performed as molecular diagnostic tools to aid in the final risk and diagnosis of PML/JCPyV infection, particularly in Acquired Immunodeficiency Syndrome (AIDS) and Multiple Sclerosis (MS) cases before or/and after initiating potent immunosuppression therapies [8,10,11]. Yet, the literature reveals that false-positive and false-negative molecular test results for JCPyV can occur; such instances raise concerns about potential misdiagnosis and further management [12,13].

In this review, biology, pathobiology and epidemiology of JCPyV and PML are discussed, and the diagnosis of PML is reviewed according to clinical manifestations, imaging features and laboratory data. Finally, an expert opinion is thoroughly presented demystifying JCPyV and the so-called PML, giving rise to further questions towards the topic.

## 2. Biology and Pathobiology of JCPyV and PML

### 2.1. Biochemical Characteristics of JCPyV

The ubiquitous JCPyV is a human polyomavirus (formerly known as papovavirus) that belongs to the Polyomaviridae family, where also BK virus (Human Polyomavirus 1), Simian Vacuolating virus 40 (SV40) and the more recent Merkel Cell Polyomavirus (MCPyV) are included. It was first supposed to be the etiologic agent of PML in a case of Hodgkin’s disease; actually, John Cunningham was the name of this patient [2,3,4,5]. An electron micrograph of potent JCPyV virions in the host nucleus has shown icosahedral structures with a diameter of approximately 40 nm or as filamentous forms forming clusters mainly in the inner nucleus periphery [14]. Regarding the initial 1971 report, virions were observed in the cytoplasm, in the nucleus, or in both, and they were mostly arranged in crystals, having a mean center-to-center distance of particles of 39 nm, while cytoplasm virions were round and these were found individually, collected in inclusion-like clusters within tubular membranes or lined up along membrane-bounded vacuoles [2].

It is believed that JCPyV has a circular double-stranded DNA in a small non-enveloped icosahedral capsid, and its genome has early and late coding sequences, being separated by a regulatory region, and also, that the non-coding control region (Transcription Control Region; TCR) lies between these early and late coding regions [14,15]. The early coding region is considered to encode multifunctional regulatory proteins and some host cell transformation proteins, the large T antigen (LTAg) and its splicing variants (T’_135_, T’_136_, and T’_165_) and a small t antigen (stAg), but it seems ambiguous whether the small t antigen is a different antigen or a splicing variant of LTAg, whereas the late coding region is supposed to encode the capsid Viral Proteins (VP1, VP2 and VP3) and a small regulatory accessory protein called Agnoprotein (Agno), which possibly performs various roles including viral replication and transcription, cell cycle arrest deregulation and downregulation, viroporin, and acting as the new virion’s transport out from the nucleus without being packaged into its structure [14,15]. Also, Agno may bind various cellular factors, such as p53, YB-1, Ku70, FEZ1, HP1α, PP2A, AP-3, PCNA, and α-SNAP, as well as LT and stAg. The VPs are potentially pivotal for the life cycle events including the host’s cellular receptor attachment, adsorption and penetration. The TCR is supposed to be included in the regulatory region and possibly contains the promoters and enhancer elements for the origin of viral replication and the expression of some early and late genes, as well as some binding sites for various transcriptional factors including a unique NF-kB site, C/EBPβ, NFAT4, Rad51, NF-1, SP1 and others, and also, the TCR contains highly divergent sequences amongst the viral isolates which determine JCPyV tropism and pathogenic effects [14,15].

Based on the structure of the TCR of JCPyV, the two known variants are Mad-1 and Archetype, where the first variant is the prototype strain that was held responsible for the first JCPyV found in PML patients reported in 1971 and has been used for most in vitro and in vivo studies of the virus; paradoxically, the Archetype is the most abundant strain in the environment and it is the transmissible form amongst individuals, and also, it has been said that this form converts to the neurotropic Mad-1 and other strains [15,16,17]. The TCR that determines the two strains is believed to be the most variable region of the JCPyV genome. The Mad-1 strain is considered to have a genome length of 5130 bps and it shares approximately 70% similarity to the genome sequence of SV40 and BK virus, while the genome also shows a similar tripartite organization with some early and late coding regions and a regulatory region [14]. There are likely to exist at least 14 subtypes of JCPyV linked to different human populations; types 3 and 6 are found in Africans, type 7A in southeast Asians, and types 1 and 4 in Europeans, who are believed to be responsible for the initial appearance of JCPyV [18,19].

### 2.2. Life Cycle and Presence of JCPyV

Literature data on JCPyV that were obtained after its isolation from latent sites such as the kidneys support the belief that the JCPyV genome is usually called Archetype, compared to the genome isolated from reactivation sites, e.g. the CNS and particularly the Cerebrospinal Fluid (CSF), where possible mutations have resulted in neurotropism [20]. The host range of polyomaviruses in culture is somewhat limited to particular cells, according to historical data. This constraint is exacerbated in the case of JCPyV, which can replicate inefficiently in human primary B lymphocytes, embryonic kidneys, amnion, transitional epithelial, Schwann and adult brain cells, and also in glial and B cell lines, whereas a significant replication has been shown in tonsillar stromal cells, yet propagation is most efficient in primary human fetal glial cells or transformants derived from them, in which the lytic cycle is delayed and virus yields are low [21]. Within the human brain, JCPyV has been found in glial cells, astrocytes, oligodendrocytes, cortical neurons and cerebellar granule cell neurons, and it has been found in both PML, in concomitant PML and cerebral neoplasm and in the absence of PML lesions, such as tumor brain lesions [22]. In fact, the term polyoma refers to the capacity of these viruses to generate tumors. It is derived from the Greek words *poly*, which means many, and *oma*, which means tumors [16].

Although little evidence of JCPyV saliva detection have been demonstrated, the first JCPyV infection is thought to occur in tonsillar tissue due to the frequency with which viruses are transferred by the ororespiratory route [22]. JCPyV transmission appears to occur within the same family, and tonsils are thought to be the first site of transmission that leads to additional hematogenous dissemination, even though the virus is frequently found in urine specimens from both healthy and immunocompromised people, indicating urine as the major source of transmission [15]. The viral life cycle starts after VP1 and pentasaccharide NeuNAc-α2,6-Gal-β1,4-GlcNAc-β1,3-Gal-β1,4-Glc interaction, and then through interaction with α-2,3-linked sialic acid, a binding that allows for JCPyV navigation in the cytoplasm via various cellular compartments before reaching the nucleus, where viral transcription precedes viral DNA replication since the early viral gene products LTAg, stAg, and splicing variants of LTAg are crucial for the beginning of the lytic cycle and other related developments [15]. The multifunctional, multidomain LTAg binds directly to the viral origin of replication, causing the transcription to switch from early to late. It also interacts with cellular modulators such as pRB and p53 through its multiple domains to control multiple cellular processes, thereby promoting cell cycle progression [15].

Additional evidence reveals that JCPyV can penetrate glial cells using both α-2,3- and α-2,6-linked sialic acids. Following this, each linkage is restored by linkage-specific sialyltransferases, suggesting that both α-2,3- and α-2,6-linked sialic acids are essential for JCPyV infection; these sialyltransferases were specific for the addition of carbohydrate to N-linked glycoproteins. Following complete restoration of the infection, it was hypothesized that an N-linked glycoprotein containing either α-2,3- or α-2,6-linked sialic acid was capable of infection [23]. However, α-2,6-linked sialic acids are most likely to be the primary means of cell entry for JCPyV. Weak interactions between JCPyV and α-2,3-linked sialic acids may also be involved in infection, though it is unclear which direct receptor molecule JCPyV favors [24]. It has been demonstrated that the viral DNA extracted from CSF samples of PML cases codes for a variety of VP1 alterations that are responsible for the binding of sialic acids; however, it is not evident whether these mutations take place in the PML individuals’ CNS or prior to JCPyV brain entry. According to reports, renal and colonic epithelial cells, lymphocytes, and glial cells are rich in sialic acid receptors. This information is meant to explain why JCPyV is primarily detected in these organs [25]. Furthermore, it is thought that JCPyV needs the serotonin receptor 5HT2AR in order to infect glial cells. Although ganglioside GT1b has also been found to function as a receptor for the virus, clathrin-dependent endocytosis is usually thought to be the method by which the virus enters the cells [24].

It has been suggested that the major receptor for the virus is the Lactoseries Tetrasaccharide C (LSTc) part from the possible receptor of JCPyV, which is paradoxically not expressed in oligodendrocytes or astrocytes found in the human brain, and also the virus does not bind to such cells. So again, one could argue that the receptor-based cell entrance is impossible, and the virus may come to exist in the cells through another method; maybe the genome is to blame. However, a study has provided evidence that JCPyV is packaged in extracellular vehicles from the infected cells, and also that the further infection of target cells via the vehicle-related virus occurs regardless of the LSTs and also it is non-neutralized by antisera against it. This study aims to provide the initial finding that a polyomavirus utilized extracellular vesicles for its transmission [26]. Other data support the understanding that various overlapping signaling pathways control the genesis-related mechanisms for these JCPyV extracellular vesicles [27].

Also, JCPyV is believed to spread to secondary replication sites including the kidneys and B cells of the bone marrow, where a lifelong persistent infection is possible [22]. Moreover, it appears that even in cases where kidney infection is asymptomatic, there may be periodic viral shedding because the virus can be found in urine specimens from healthy people and can live there for an extended period of time with full immune function. However, following a period of severe immunosuppression, JCPyV can reactivate and spread to the central nervous system (CNS), potentially via B lymphocytes after crossing the Blood-Brain Barrier (BBB) [22]. JCPyV is permissive in bone marrow and peripheral blood B-lymphocytes, suggesting that viral spread after initial replication can occur through a hematogenous route or crossing the BBB via these cells [15]. Nevertheless, it is unclear if JCPyV is able to spread into the CNS or if a latent infection there can locally reactivate it [15]. Furthermore, since horizontal transfer from mother to fetus has also been reported, it is unknown if the virus can already exist in fetus cells before or after CNS formation [28]. Additional information points to a potential carcinogenic function for JCPyV, as evidenced by the fact that its genome is expressed in a variety of tumor tissues, including colon cancer [16]. Thus, one could argue that this possible virus exists in every human cell—a hypothesis that needs to be proven!

### 2.3. Neuropathobiology of PML

Currently, it seems unknown whether JCPyV VP1 mutations cause PML or not, but it is believed that this virus enters into the cells via clathrin-dependent endocytosis by using α-2,3- or α-2,6-linked sialic acids or 5HT_2A_R or the ganglioside GT1b [24,25].

Despite the fact that JCPyV neuropathology affects the central nervous system, seroepidemiological research shows that the majority of people worldwide are either latently or transiently infected with the virus, with population heterogeneity even in non-industrialized areas, and evidence that seropositivity rises with age. However, it was previously discussed that NCCR rearrangements may be associated with viral reactivation and the presence of the so-called JCPyV in the CNS [25].

The first PML incidences were reported in 1958 in patients with underlying chronic lymphocytic leukemia and Hodgkin’s lymphoma [1]. In 1959, suspicions regarding a viral etiology in PML were raised by discoveries of inclusion bodies (particles resembling those of related polyomaviruses) in the nuclei of injured oligodendrocytes. However, an unusual viral infection could perhaps account for the pattern of lesions [29,30].

Prior to 1981, when the AIDS epidemic stigmatized the country, there were only 238 documented cases of PML in the literature. These cases included recipients of organ transplants, patients with hematological malignancies, lymphoproliferative diseases, and cases of chronic inflammatory disorders after immunosuppressive therapy. These days, AIDS is the most common underlying medical condition in PML patients, followed by cancer and some MS patients taking a particular immunosuppressive medication [31]. The histology of PML-affected brain tissue has demonstrated a productive infection of macroglial cells, while the presence of unusual giant astrocytes and large oligodendrocytes with enlarged nuclei and multiple intranuclear eosinophilic inclusion bodies, along with subcortical demyelination, is thought to indicate an active viral replication [31].

PML neuropathology—as seen in the initial reports—is described by the development of several white matter plaques in the brain stem, basal ganglia and thalamus, cerebral hemispheres, and cerebellum [1]. These days, PML is divided into categories that are associated with HIV and those that are not. It typically affects the cerebral white matter, brainstem, and cerebellum, although it hardly ever affects the spinal cord. High rates of HIV-positive PML cases are thought to result from a number of factors, such as the presence of CNS HIV, which may either directly or indirectly contribute to PML neuropathogenesis, CD4+ T-cell loss with compromised CNS immune surveillance, and the activation of CD8+ Cytotoxic T Lymphocyte (CTL) responses with the destruction of infected oligodendrocytes [32].

Brainstem and cerebellar involvement are not exceptionally uncommon in non-HIV cases, however PML lesions confined to the cerebellum and brainstem are less common. Non-HIV and non-malignancy-associated PML types can affect different brain regions [33]. These findings imply that the clinical prognosis of non-HIV PML in the cerebellum and brainstem forms may depend on the patient’s overall health status in relation to comorbid conditions, and that comorbid chronic renal failure may have a poor prognosis in such cases. Additionally, cases of non-HIV infratentorial-onset PML may have compromised status due to a variety of underlying diseases, including malignant lymphoma, idiopathic CD4 lymphocytopenia, and chronic renal failure [33]. For example, in a 52-year-old patient with a history of severe chronic heart failure brought on by myocardial infarction, diabetes mellitus, hypertension, dyslipidemia, and chronic kidney disease (CKD), an autopsy revealed a progressive white matter lesion in the right frontoparietal lobe involving the precentral gyrus, large demyelinated lesions involving the fusion of several small lesions, a central lesion involving deep gray matters such as the putamen and thalamus, lesions in the infratentorial brainstem and cerebellum, demyelination in the pontine basilar region, and in cerebellar white matter that was contiguous via middle cerebellar peduncles. Satellite lesions were dispersed throughout the brain, suggesting that PML lesions most likely develop following initiation via extension/expansion and fusion [34]. 

Because the initial infection site, the tissue or region of viral latency, and the circumstances under which JCPyV penetrates the brain are thought to be incompletely known, the pathophysiology of PML remains unclear. However, the presence of transcription factors that bind to the viral TCR, immunodeficiency, genetic predisposition, and changes in the viral TCR that increase viral transcription and replication all contribute to the onset of PML caused by the so-called JCPyV [32]. For instance, some data support a possible predisposition for PML amongst those with type 0 red blood cells, and other data reveal that in HIV-infected PML patients the TCR shows multiple upstream Tat-responsive element (up-TAR) duplications, which is also constantly found at the 5′-end of HIV-1 mRNAs, and this region is involved in HIV Tat induction of the viral late promoter [35,36]. Additionally, it is believed that JCPyV infection can affect bone marrow B-lymphocytes and CD34+ hematopoietic precursor cells, which are thought to be the virus’ reservoir and source of TCR rearrangement and brain transmission. This is especially true when the number of these cells is elevated in peripheral blood circulation [32].

Additionally, it is thought that the histology of PML lesions is characterized by modifications to oligodendroglial nuclei alterations, demyelination, and abnormalities to astrocytes; BBB disruption, lymphocyte infiltration, and edema are typically absent [9,37]. However, in contrast to classic PML, lesions resulting from the Immune Reconstitution Inflammatory Syndrome (IRIS) may exhibit contrast enhancement on MRI due to localized BBB breakdown and inflammation. This may be linked to brain edema, swelling, and mass effect, which in the most extreme cases can result in brain herniation and death [37]. Moreover, CTLs that identify and eliminate JCPyV-infected oligodendrocytes infiltrate PML-IRIS brain lesions, which not only prevents the virus from spreading but also results in demyelination and oligodendrocyte cell death. In fact, PML-IRIS lesion neuroimaging demonstrates contrast enhancement, mass effect, and interstitial edema [32]. But even in cases of hematological malignancies, these findings may differ across possible other prior medical conditions and the drugs that may be used to treat them [38,39,40].

Some reports on MS IRIS natalizumab-associated PML patients reveal extensive CD8+-dominated T cell infiltrates and numerous macrophages within lesions in nondemyelinated white and grey matter as well, whereas no JCPyV-infected cells have been found—a fact that could indicate HIV-PML derived IRIS [41]. Moreover, higher plasma cell counts have been reported in comparison to PML without MS and typical MS lesions [41]. Thus, although PML was thought to be a non-inflammatory illness that specifically targeted astrocytes and oligodendrocytes in the white matter of people in immunosuppressive states, it can manifest during an immune reconstitution immune system infection (IRIS) that affects a range of phenotypes, from those with mild-to-moderate immunosuppression to those who are completely immunocompromised or on immunomodulation medication. Glial cells thought to be JCPyV-infected are often found at the gray matter–white matter junction or directly into the gray matter, where they cause demyelination in cortical and subcortical regions [38,42]. Additionally, it has been said that may the virus infect neurons, thus resulting into both JCPyV granule cell neuronopathy or/and JCPyV encephalopathy [42].

With the exception of PML, JCPyV-suspected encephalopathy has been recorded in cases of infected gray matter cortical pyramidal neurons, while JCPyV-positive viral meningitis has been described in cases when no other viral infections are present [22]. Although kidney JCPyV infection is generally not thought to cause clinical illness, this virus has been linked to polyomavirus-associated nephropathy on its own. Lastly, this virus has been classified as Group 2B for “potentially carcinogenic to humans” due to potential interactions with colon cancer, though a direct correlation has not yet been established [22].

## 3. Epidemiology of JCPyV and PML

It seems difficult to estimate the period of the primary infection because JCPyV is believed to cause an initial asymptomatic infection; yet, for the majority, infection is more likely to occur in childhood since about one in ten children < 6 years old are anti-JCPyV antibodies seropositive, with these rates to increase during adult life and reach up to 70% and more, at a global level [15]. However, the reported seroprevalence varies from 39% to 91% and it seems to depend on assay methodology as well as the population studied [43].

JCPyV infection can vary a lot, and a study on 7724 MS cases from 10 countries showed that seropositivity was lower in females (55.3%) than males (61.6%) and lower in cases without current/prior immunosuppressant use (56.6% vs. 63.7%), and Portugal, Austria and The Netherlands showed the highest seroprevalence compared to Australia and UK which had the lowest, while no significant differences in anti-JCPyV antibody titers were linked to MS characteristics, including duration and type of MS and number and duration of MS medications [44]. Another study on more than 16,000 MS cases showed a significant JCPyV seropositivity heterogeneity, ranging from 40% to 80% [44]. Except from MS, CSF samples from cases with neuroinvasive diseases like febrile headache, meningitis and encephalitis were not found to be positive for the viral DNA, but males and the elderly were more likely to be seropositive [45]. Regarding HIV infection, JCPyV DNA in urine samples was detected in 80.2% of HIV-infected individuals and in 53.8% of healthy individuals [46]. Additionally, up to now, no clear prospective studies supporting a link between JCPyV and cancer in humans have been conducted, but various studies have been conducted to establish the association between JCPyV seropositivity and specific human tumor types, like colorectal cancer, lymphoma, central nervous system tumors, esophageal carcinoma, carcinoma of the bladder, and prostate cancer [47]. Furthermore, the virus seems to be present in gastrointestinal tract mucosa of immunosuppressed patients with cancer and there is a relative risk of 10.4 (prevalence 35.3%) for organ transplant recipients to be positive in JCPyV nucleic acid tests, yet the risk for the first cases is lower than for the second ones [47].

A study on 584 patients with incident PML in which 7% had an IRIS, revealed that predisposing conditions were HIV/AIDS (43.7%), hematological malignancies (21.9%), chronic inflammatory diseases (20.2%), solid organ transplantation (4.3%), solid neoplasm (4.1%) and primary immune deficiency (1.5%), and the 1-year mortality rate was 38.2%, whereas some factors independently linked to death were older age, male gender and predisposing immunosuppressive states, with the highest risk for solid neoplasms (adjusted hazard ratio 4.34 in multivariate analysis), followed by hematological malignancies and HIV/AIDS, compared with chronic inflammatory diseases [48].

There have been various case reports on PML and its manifestations in individuals with different preexisting medical conditions. For instance, PML has been associated with chemotherapy-induced lymphocytopenia in solid tumors and it has been recorded in a case with follicular lymphoma treated with anti-CD20 monoclonal antibodies, and another spontaneous report was on a patient undergoing treatment for advanced ductal breast carcinoma and systemic sclerosis who developed the disease, whereas it has been discussed that atypical findings should not exclude diagnosis in cases that are not likely to have classical PML [49,50,51,52]. PML has been documented in people with various autoimmune-supposed conditions—apart from MS—who follow immunomodulation therapy, like Systemic Lupus Erythematosus (SLE) in parallel with cancer complications or not, Rheumatoid Arthritis (RA), ANCA-associated vasculitis, autoimmune hepatitis, myositis, ulcerative colitis, lupus nephritis, ankylosing spondylitis and Crohn’s disease [53,54,55,56,57,58,59,60,61,62]. Also, PML has possibly been evident in a case with Coronavirus Disease 2019 (COVID-19) as well as in a patient with Hepatitis B virus-induced CD4 lymphocytopenia, while the disease may occur in the context of systemic sarcoidosis without immunosuppression in which it can even be the first sign, and it can initially be mistaken for neurosarcoidosis or other complications of sarcoidosis [63,64,65]. Also, divergent cases of chronic physiologic diseases/disorders under various therapies can spontaneously express PML, like Chronic Liver Disease (CLD), Chronic Kidney Disease (CKD) and other issues [66,67].

## 4. Diagnosis of PML

The diagnosis of PML has evolved notably after its emergence in 1958 [1]. At a first glance, PML diagnosis was predicated on brain histopathology due to the absence of clinical, laboratory, or radiographic data that could unequivocally establish the diagnosis, and histopathology was characterized by the classic triad of demyelination, bizarre astrocytes and oligodendroglial nuclear inclusions [1].

More than a decade ago, it was declared that PML diagnosis seems: (i) definite, if clinical features and imaging features are compatible along with positivity in a CSF PCR test for JCPyV, (ii) probable, if a PCR test for JCPyV is positive and clinical features or imaging findings are compatible, (iii) possible, if clinical features and imaging findings are compatible but PCR is not performed/equivocal result, or the opposite, meaning a positive PCR test but no compatibility in the other ones and (iv) negative for PML/not PML, if only clinical features or imaging findings are compatible but a PCR test is negative, as seen in Table 1 [9].

Also, the diagnosis of PML histopathology supposes it as: (i) definite, if classic histopathologic triad and tissue PCR test for JCPyV and immunohistochemistry/electron microscopy are all positive or even if only the last one or only the positivity in viral DNA is absent, (ii) probable, if there is positivity in classic histopathologic triad but the others are absent (iii) possible, if immunohistochemistry/electron microscopy is positive but the others are absent, and (iv) negative for PML/not PML, if all are negative and a DNA test is not performed, as seen in Table 2 [9].

As a result, there are two ways to diagnose PML; its diagnosis can be secured with tissue or, as commonly, with clinical or radiologic evidence along with the presence of viral DNA in CSF.

Yet, the spectrum of JCPyV tropism can spontaneously involve the meninges (JCPyV meningitis), pyramidal cells (JCPyV encephalopathy), or cerebellar granule cells, and GCN may happen with or without concomitant cerebellum or brain demyelination, and infection may lead to a loss of granule cell neurons as well as cerebellar atrophy [68].

### 4.1. Clinical Manifestations of PML

In the pre-AIDS era, about 1/3 of PML patients reported cognitive/behavioral issues and similar percentages reported motor weakness as well as visual deficits, while about 1/5 of them had speech or language disorders; AIDS-associated PML shares similar rates in cognitive/behavioral issues, but less than half of the cases show motor weaknesses and speech/language disorders, and about 1/3 of them show headaches as well as gait abnormalities and incoordination, whereas in natalizumab-associated PML, more than half of the cases are more likely to have cognitive/behavioral issues, and less than half of them can have motor weaknesses and visual deficits [9]. However, such symptomatology in the last scenario of natalizumab-associated PML is highly affected by the preexistent MS lesions and subsequent pathognomonics.

The disease seems not to affect the peripheral nervous system, and spinal cord as well as optic nerve involvement are believed to be rare [9,69,70]. In rare cases, PML may be the first manifestation of AIDS [71]. The disease can even be manifested as unilateral sensorineural hearing loss, and monoparesis or hemiparesis and dysphasia can be some other initial clinical symptoms [72,73]. Some other very old and extremely random PML reports for cases with no known immunosuppression reveal that initial presentations of the disease may include decreased arm/leg power, nervousness, fatigue, lethargy or paranoia [74,75].

It should be noted that microscopic pathological alterations can take place before imaging changes are observed which may precede clinical symptoms [76]. The earliest pathological lesions are small with infected glial cells and microglial/macrophage activation, with subsequent oligodendrocyte loss and demyelination. Reversely, asymptomatic people with PML can have small lesions featuring glial changes with or without demyelination, which are supposed to be the earliest pathological characteristics; interestingly, asymptomatics may also have totally developed PML lesions indistinguishable from those found in a symptomatic PML patient [77].

It must also be highlighted that clinical symptomatology depends on the extent of the lesions of each case and the potential preexistent lesions, the rates that these lesions progress, as well as other parameters like age, sex, diet, medical history and possible medications, daily routine and lifestyle and other critical evidence that has a direct impact on symptoms and signs of a disease.

### 4.2. Imaging Features of PML

A brain MRI is necessary for PML diagnosis and surveillance. This multisequence image acquisition strategy, which depends on the clinical context, maximizes the usefulness of each sequence in terms of identifying specific PML lesions. A shortened procedure without contrast-enhanced T1-weighted sequences has been suggested for pharmacovigilance (i.e., for monoclonal antibody therapy), but it does include Fluid-Attenuated Inversion Recovery (T2-FLAIR), T2-weighted, and Diffusion-Weighted Imaging (DWI) [78]. Contrast-enhanced T1-weighted evidence appears to be necessary, especially for monitoring purposes, to detect PML-IRIS; however, Magnetic Resonance Imaging (MRI) cannot distinguish between PML-IRIS, ongoing PML, and MS exacerbations; even in cases of SLE, MRI can be a general diagnostic tool, encompassing diseases related to the disease like cerebral vasculitis, infections, and malignant disorders such as lymphoma [79,80].

It seems that T2-FLAIR images reveal the extent of PML lesions, and, compared to classic PML lesions, inflammatory PML lesions typically may have fewer infected cells. Intralesional vacuoles may be seen in T2-weighted turbo/fast spin echo sequences. In inflammatory PML, contrast-enhanced T1-weighted images often demonstrate enhancement, while in advanced stages, T1-weighted signal intensity may be minimal. Furthermore, in classical PML, high DWI signal intensity indicates actively inflammatory regions where viral replication causes oligodendrocyte swelling. The lesions may exhibit variable ADC values, but diffusion is rarely noticeably constrained [68]. Nonetheless, the disease’s imaging pattern may differ depending on the kind of immunosuppression that causes it, particularly when comparing PML associated with HIV to PML associated with monoclonal antibodies. Furthermore, conventional T2-weighted images can describe lesion size and location (commonly seen in the frontal and parietooccipital lobes and rarely in the temporal lobes, the basal ganglia region, and the posterior fossa), as well as show lesion borders that are sharp near the cortex but blurred near the white matter, perilesional nodules, grey matter involvement, and possible intralesional vacuoles, even if T2-FLAIR, and especially the 3D FLAIR, is sensitive enough to detect PML lesions, which can be unilobar, multilobar, or widespread [68]. After diagnosis, T2-weighted sequences seem valuable to estimate the progression of the disease, and lesions tend to expand continuously in cases without immune reconstitution; yet, in cases with partial or complete immune reconstitution, lesions can expand slowly for weeks to months and PML-IRIS may be subsequent, whereas in PML-IRIS, a rapid expansion can be seen in lesions that is accompanied by inflammation signs like perilesional oedema, mass effect, enlargement of perivascular spaces, and contrast enhancement [81,82].

Though information about the presence of a central vein in PML lesions appears to be lacking, making such differentiation appears unlikely. This raises concerns because the spinal cord and optic nerves are rarely affected in the disease. It is also possible that other comorbidities or diagnoses can be distinguished from PML [83]. Moreover, a number of tiny punctate lesions outside the primary PML lesions may be visible on T2-weighted sequences, and some of them may exhibit contrast enhancement. Despite the lack of clarity around their histology, these lesions could signify inflammation in the perivascular spaces [84]. Nevertheless, the same parameters that were previously reported to have an impact on the symptomatology of the disease, affect the time interval between follow-up scans too.

A further decrease in T1-weighted signal intensity that approaches that of CSF is attributed to disease progression. However, the loss of reversion to T1-weighted isointensity and the subsequent encephalomalacia may illustrate prominent tissue destruction and no important remyelination. Initially, PML lesions are isointense or mildly hypointense on unenhanced T1-weighted images, a fact that reflects the early stages of the disease [85]. The basis for classical PML is gadolinium enhancement, which was first measured in HIV/AIDS patients with uncommon contrast enhancement. However, as was previously mentioned, the inflammatory condition is also frequently observed in patients who are not on HAART and in PML linked to natalizumab. A more common occurrence in natalizumab-associated PML is contrast enhancement, which can be seen even in asymptomatic and early lesions. This contrast enhancement often reflects a perivascular distribution pattern, such as punctate lesions, and may reveal incomplete immune suppression; histological evidence of T-cell infiltration in inflammatory PML may support the idea that partial immune re-constitution plays a role in this imaging feature [86]. However, as previously mentioned, MRI is not capable of differentiating reliably between desirable inflammation and PML-IRIS associated inflammation [68].

Furthermore, in Susceptibility-Weighted MRI (SWI) some compounds that affect the local magnetic field, like calcium, iron, myelin, and blood derivatives, modify the MRI signal phase, and slightly modify its magnitude (T_2_*w images), and PML lesions can be seen as a paramagnetic leukocortical band regardless of the predisposing factor that can thicken and spread continuously along the juxtacortical white matter; yet, this leukocortical band can be seen in other disease states such as stroke and encephalitis [87].

### 4.3. Laboratory Data of PML/JCPyV

#### 4.3.1. Blood and CSF Biomarkers

It was discussed in previous paragraphs that an ABO blood group—referred to as a 0 group—is possibly linked to a higher risk for PML [33]. Apart from JCPyV DNA positivity which will be discussed in the following section, some common infection characteristics are evident in the literature. A study on HIV-infected individuals who manifested PML, and where more than half of them were treated with HAART, revealed that almost all patients had a nadir CD4^+^ cell count < 200 cells/µL, very few patients had a fever and leukocytosis, but about half of them had an elevated sedimentation rate or C-reactive protein level; CD4+ cell count > 50 cells/µL at PML diagnosis was significantly linked to reduced mortality [88]. A similar cell count was reported for PML-IRIS, while the vast majority of them had a median of HIV viral load ≤ 200 copies/mL [89]. In natalizumab-associated PML, IFN-γ, TNF, IL-2 and IL-10 production is evident, and a transient increase in IL-10-producing cells after the initiation of natalizumab and high frequency in individuals with PML has been noted [90]. In some old data describing spontaneous PML cases even without immunosuppression, it was shown that common laboratory results were normal [74,75]. A study showed that lesions of PML-IRIS patients showed significantly higher choline/creatine (*p* = 0.0001), myoinositol/creatine (*p* = 0.02), lipid/creatine (*p* < 0.0001), and lactate/creatine (*p* = 0.002) ratios and lower N-acetylaspartate/creatine (*p* = 0.02) ratios than those with PML but without IRIS [91]. Individuals who carry HIV and who are diagnosed with PML may have significantly elevated TNF-α and TNFR1 in brain samples, and also, the disease has been associated with the specific loss of cellular L-selectin on CD4+ cells [92,93]. Moreover, L-selectin seems to be an ambiguous biomarker in natalizumab-associated PML MS patients whereas matrix metalloproteinase 9 decrease may be a risk for PML in the same group, and also, serum neurofilaments have been reported to be elevated in pre-PML and PML in MS cases [94,95,96,97]. Some other immunological biomarkers like the leukocyte cell membrane markers named CD49d, CD11a, and CD62L, the detection of circulating JCPyV-specific activated T effector memory cells and genetic screening have been proposed for PML [98].

Concerning CSF, apart from the viral DNA, in HIV-infected PML patients, pleocytosis was not common but increased protein levels could be possible in almost half of the patients, yet abnormal decreases in glycose level may not be observed [88]. For the IRIS-PML cases, a study reported that the total leukocyte count median was 1 cell/mm^3^ (0–2), while the median glucose and protein levels were 61 mg/dL (50–69) and 36 mg/dL (25–41), respectively [89]. A study on CSF analysis in 108 patients with PML demonstrated that compared to the routine CSF parameters, 22% had an elevated cell count and 35% showed an increased Qalbumin indicating a disturbed blood-CSF-barrier; the mean lactate content was 1.64 mmol/L, whereby 6% presented with elevated lactate levels; PML patients showed an increased cell count—mainly driven by HIV-PML cases, among whom a significantly higher proportion had a disturbed blood-CSF-barrier function [99]. Moreover, herpesviruses such as herpes simplex virus (HSV), varicella-zoster virus (VZV), cytomegalovirus (CMV), Epstein-Barr virus (EBV), and human herpesvirus 6 (HHV-6), are occasionally detected in the CSF from PML patients and immunocompromised individuals who are suspected of having the disease [100].

#### 4.3.2. Molecular Diagnosis of JCPyV Nucleic Acid

In general, procedures involved in the diagnosis of JCPyV CNS infection range from the detection of viral products in biopsy material to the demonstration of viral genes in CSF by PCR. Nowadays, the current golden method for laboratory diagnosis of PML is considered to be JCPyV nucleic acid identification in probable cases, via PCR assay in CSF clinical specimens [101]. Real-time PCR is the technique of collecting data throughout the PCR process as it occurs, and rPCR can amplify DNA, or, when preceded by a reverse transcription, RNA [102]. The JCPyV has a small circular, double-stranded DNA genome, thus PCR assay does not require reverse transcription before being performed. Commonly, PCR reveals the presence of JCPyV DNA in the peripheral blood lymphocytes, but except from blood samples, this technology is applied in urine and CSF clinical specimens, too. With an 81–94% sensitivity and a 95–100% specificity, PCR was the golden standard for molecular diagnosis; however, the sensitivity has significantly been reduced after the introduction of HAART therapy, because of the low copy number, due to the reconstitution of the immune system [103]. Indeed, CSF analysis in proven PML cases either with or without HAART history can show very low or zero JCPyV DNA levels (<500 copies/mL) in more than half patients, a fact that highlights the high rates of a false-negative test result for CSF JCPyV DNA [104]. Yet, further PCR-based and more sensitive and specific assays have been established; for instance, a multiplex quantitative PCR was performed to monitor JCPyV NCRR rearrangement at the same time of quantification, and to discriminate between virulent and non-virulent NCRR rearrangements [105].

False PCR test results can occur due to erroneous test administration, untutored use and deviation from test kit’s interim guidance. False-positive PCR test results can occur due to poorly experienced laboratory personnel and handling or sampling contaminations, and technical issues (i.e., issues in probes’ fluorescence) [7]. False-positive test results could also occur when only one JCPyV genetic loci is detected, compared to two or more gene detections in some PCR assays. The Cycle threshold (Ct) value is defined as the number of cycles required for the fluorescent signal to cross the threshold, so as to obtain a PCR result. The viral load presented in the sample is inversely related to the Ct value, and high viral loads lead to a low Ct, thus a positive result, and low viral loads lead to a high Ct, therefore a negative result, according to Ct cut-off value of each kit’s interim guidance. The Ct value can affect the PCR test result, and false results can occur since various PCR test kits may have different Ct values, leading in different test results [106,107]. False-positivity could be eliminated by reducing the Ct cut-off value, but the reduction of the Ct cut-off value may give rise to false-negative test results [108]. Thus, in general, the unjustified increase in cut-off values can be important for increasing the specificity of a given assay, but this may jeopardies test’s sensitivity and clinically relevant diagnosis. Another important issue is that PCR assays detect genetic loci, and this translates to a positive or negative test result. Nevertheless, a positive test result does not mean that the detected JCPyV gene was from an active JCPyV; inactive or residual genes can also lead to positivity. In other words, JCPyV DNA can be present in many human brain samples from patients without PML [109]. This issue makes sense, considering the fact that viremia can exist before PML clinical symptoms and radiologic data take place, or reversely, in the final PML stages, due to viral clearance. Finally, regarding false-positivity, it is obvious that PCR kit manuals report some other pathogens that they do not detect, since they are highly sensitive, so as to achieve a valid positive test result. Also, it cannot be excluded that other pathogens not inserted in some PCR kit manuals could cross-react, thus giving a false-positive test result.

False-negative PCR test results also seem to be frequent [13,110]. There are several reasons for false-negativity in PCR, such as deficient sampling or low sample volume, destroyed/expired reagents or JCPyV nucleic acid degradation until the specimen arrives at the laboratory [7,102,109]. Another important parameter to take into account for a potential false-negative PCR test result may be the lack of spontaneous dissemination of JCPyV into the subarachnoid area. The Load of Detection score (LoD score) also affects PCR test results, as most kits’ interim guidance report the lowest JCPyV load that the assay can detect [110]. For example, the ultrasensitive TaqMan real-time PCR assay (available at National Institutes of Health and other international laboratories) is able to detect fewer viral loads > 10 DNA copies/mL, whereas other readily available quantitative real-time PCR techniques have a reported threshold of >50 DNA copies/Ml, as reported in the related study [110]. Viral mutations in the targeted genetic loci can also result in false-negative test results. Additionally, a bad DNA extraction could result in false negativity due to PCR inhibitors, as several PCR test kits’ interim guidance mention in their limitations [111]. Ct value can also affect the final result, since different methods may have different Ct cut-off values, leading to different results [106,108]. Usually, PCR assays include a heterologous amplification system (internal control) to identify possible inhibition. Despite that fact, inhibitors are still present in kits’ interim guidance [109]. PCR inhibitors act on one or more essential stages of PCR testing procedure, from nucleic acid binding, DNA polymerase inhibition, or ionic buffer alteration, all of which can increase the Ct value and give rise to false-negative test reports. There have been several substances that can inhibit PCR assay reported, including mainly IgG, hemoglobin, lactoferrin, heparin, myoglobin, urea, bile salts, bilirubin, free radicals, specific metal ions and ethanol [112,113,114,115,116]. In this manner, CSF samples that could include such substances may be at high risk for a false-negative PCR test result. For instance, the presence of oligoclonal bands (OBs) in the CSF samples of cases with CNS inflammation, due to infection or MS, is evident [117]. Cases with excessive Obs in their CSF samples could show a false-negative PCR test result, in a poorly performed assay. A case report has been reported with false-negative PCR test results, in a MS case with oligoclonal bands, and some other CSF findings, and, in this specific case, the false results could potentially be predicted [13]. In similar manner, individuals with other preexistent neurological disorders and neuroinflammation that can result in saturated clinical samples may have false results. Also, viscous or bloody CSF samples are at high risk for a false result. For example, a clinical CSF specimen from a case with an acute Subarachnoid Haemorrhage (SAH) could show a false-negative PCR test result if the assay is poorly performed [118,119]. Specific drugs penetrate through the blood-CSF fluid/blood-brain barrier for the treatment of CNS infections [120]. May such drugs also have an inhibitory effect in poor PCR assays. In this manner, potential inhibitors should be identified before any PCR test result is reported. Additionally, there have been processes proposed to improve the detection of JCPyV by ultrafiltration of CSF before PCR, for the confirmation of PML [121].

#### 4.3.3. Molecular Diagnosis of JCPyV Antigens and Anti-JCPyV Antibodies

Principally, there are several factors that can affect antibody production (i.e., Immunoglobulin M (IgM) and Immunoglobulin G (IgG)), such as sex, genetics, diet, adjuvants, immunosuppressants, vaccines, etc. These factors may affect the sensitivity of an antibody detection assay [122,123].

The most relevant common serological assays are the enzyme-linked immunosorbent assay (ELISA) and chemiluminescent immunoassay (CLIA). Technical issues or assays performed by inexperienced personnel, deviations from testing protocols, testing in a window period (pre-symptomatic or post-symptomatic period), poor sampling and handling, or antibody-related parameters are common for false IgG/IgM test results [102,109]. When a patient has an early PML, IgM may not be detectable in serum/plasma, and IgM and IgG antibodies may present a sensitivity heterogeneity.

Immunoassays such as ELISA can be affected by specific exogenous administered antibodies (monoclonal antibody drugs) or endogenous antibodies (heterophile, autoantibodies, antinuclear or anti-animal) that interfere and give a falsely elevated result in one assay or a lower result in another assay, even in the same individual [124]. Human anti-animal antibodies, found in animal workers (i.e., veterinaries, breeders, or cases with chronic indoor animals), can also interfere and lead to a false serologic test result [125,126]. The presence of human anti-bovine IgG antibodies and the evidence that all human beings present autoantibodies potentially interfering in immunoassays conclude that serologic test results should be interpreted according to an individual’s overall health condition [127,128]. HIV, hepatitis, rheumatoid factor, syphilis, malaria, lupus, vasculitis, hypergammaglobulinaemia and the presence of HLA-DR antibodies have long been correlated with false serologic test results and antibody interference [7,102,109]. The most profound cause of a false-positive test is a state of hyperglobulinaemia in the serum or plasma of the individual under consideration, which is the most common cause of a false-positive test—the so-called “sticky serum”. Also, general antibody titers in one’s sample could have an impact on the final test result. Moreover, there have been some other factors that can affect a serologic test reported, such as hemolysis, icteria, lipemia, hook effect, metabolites of certain medications/foods in daily and moderate-to-high consumption (i.e., specific minerals), radioactive/fluorescent compounds, aggregated complexes and different post-translational modifications for anti-JCPyV immunoglobulins in a sample, and heterophile antibodies in general including HAAAs (due to interactions with animals and animal products—a type of these antibodies are HAMAs), anti-streptavidin/anti-ruthenium/anti-biotin antibodies, autoimmune-supposed antibodies, IgG4 that are present in every person, vaccination status, blood transfusion, transplants, maternal transfer, pregnancy, multiparous women, hemodialysis, surgery, paraproteins like the previously mentioned case of hypergammaglobulinemia but also in pseudohypophoshatemia, dyscrasia, multiple myeloma, lymphoma, Waldenstrom macroglobulinemia, amyloidosis, monoclonal gammopathy, light chain disease, cryoglobulinemia, and chronic systemic inflammation that is common in every person nowadays from a low to high extent [129]. Apart from cross-contaminations in patients’ samples, bacterial contamination of serum or plasma specimens or reagents can produce erroneous results [130]. Most immunoassays are tested for cross-reactions with other antibodies from other pathogens, so as to be valid. However, the presence of cross-reactive antibodies against shared epitopes of pathogens cannot always be excluded. Moreover, considering the fact that as new pathogens emerge, we cannot be sure if a specific immunoassay can show cross-reactions with other antibodies from other sources. Furthermore, it was previously mentioned that vaccine-induced immunity can cause false results and there is a report for a false-positive ELISA result for HIV after influenza vaccination [131]. Hence, the vaccination status of the individuals—before sampling for anti-JCPyV—should be recorded and taken into account as a likely cause of potential cross-reaction so as to lead to false test results.

Last but not least, it is obvious that high-dose biotin has emerged as a promising therapy for RRMS cases, as it promotes axonal remyelination by enhancing myelin production and by reducing axonal hypoxia through enhanced energy production [132]. However, biotin interference in immunoassays is a common event, since there are reports of false test results [133,134]. Also, biotin at various concentrations is taken as a nutrient supplement, further increasing concerns as to whether such supplementation may interfere with immunoassay testing. It is important that the FDA has issued specific warnings regarding the issue of biotin’s interference with lab test results, and in particular those related to immunoassays [135]. Also, the American Association Clinical Chemistry Academy issued new recommendations for proper testing which can minimize the risk of biotin supplementation interference with test results [136].

Finally, it is obvious that contrary to the current state of research on sandwich immunoassays, falsely elevated test results can be more frequent than falsely low test results, as most assays take into account the issue of high sensitivity rather than that of high specificity [137]. However, not only such parameters affect blood samples, but also CSF samples; MS cases may have higher anti-JCPyV antibody titers [138].

JCPyV antigen detection can be affected by similar parameters that have an impact on serologic test results, specifically those affecting ELISA and other immunological assays for antigen detection.

## 5. The Expert Opinion—Demystifying JCPyV and PML

As with all diagnostic tests, the interpretation of antibody or PCR test results for the so-called JCPyV identification should be interpreted in parallel with all clinical and laboratory findings. When the clinical presentation is probable for the supposed PML, an initial negative PCR test does not exclude the presence of the virus, while a positive PCR test does not always indicate the presence of an active disease, since it may be at the final stage (i.e., viral clearance). Potential PCR inhibitors and antibody interference should always be taken into consideration for the laboratory diagnosis of the possible virus. Such cases should be reported for the laboratory experts to perform highly sensitive testing assays. Re-testing should also be considered, as well as the alternative sample diagnosis, since a test result illustrates a random sample at a random point if time [139]. Also, the combination of PCR and ELISA in case of ambiguous data could be important. Nevertheless, since tests seem to be unreliable and error-prone, maybe more accurate diagnostic tests ought to be designed, for a precise diagnosis of all diseases. The real question for an accurate test to answer is: What should be tested for? What will be the real potential target?

Moreover, cut-off values of antibody testing may take into account the age of the individual, as anti-JCPyV antibody titers can be age-dependent [140]. Therefore, a high serologic status in a young adult could indicate a potential false antibody test result. Such cases’ antibody monitoring should be repeated, for a more accurate and precise serologic status. Cases with extra antibody load—especially from the previously reported causes—or high antibody titers in their blood samples should be monitored or even mentioned by the laboratory experts. Also, regarding biotin interference in streptavidin-biotin antibody assays, it is urgently required that MS cases stop biotin supplements before anti-JCPyV serostatus monitoring, so as not to allow for a false antibody test result. There have been several recommendations for biotin pause before sampling; it is proposed that patients who have consumed 5–10 mg biotin should wait a minimum of 8 h before blood sampling, whereas people receiving higher doses should stop biotin supplements for at least 72 h before sampling, or follow their doctor’s instruction about biotin pause and their required time [136].

In general, regarding testing assays, it is fundamental for proper diagnostic testing where assays take into account sensitivity and specificity as well as positive and negative predictive values when discussing the accuracy of tests in the clinical setting. Finally, even if PML was correlated with AIDS and MS cases treated with natalizumab, there exist many new immunomodulators and immunosuppressants in several autoimmune diseases. For instance, PML has been reported in cases treated with fingolimod or rituximab [141,142]. Yet, it may be too early for PML cases to be correlated with new immunosuppressants. In this manner, PML requires accurate and precise diagnosis.

A recent perspective discussing molecular diagnostic lessons that the current pandemic has taught the scientific community highlights that the molecular diagnostic testing needs to be improved in several ways, mainly regarding testing terminology and its role; it is not the disease but the carrier of a specific biomolecule or genetic sequence that is identified during testing [7]. Additionally, critical quantity and quality issues have been discussed, thus it seems wise to say that sensitivity and specificity are inversely related, which means as sensitivity increases, specificity decreases, and vice versa. As a result, it does not seem wise to discuss more on specificity and sensitivity, as each test has its own standards and limitations as well. Moreover, it is not scientific as such to compare sensitivity and specificity of each test type, i.e., a PCR test versus an antibody test, since each different test performs a different role in the biomolecule that is detected [7].

Yet, it is evident that JCPyV status was not such studied in the pre-immunotherapeutic era, and important literature data have been presented mostly after some AIDS and MS therapies. Previous evidence suggests that JCPyV infection occurs universally, increasing with age, varies amongst populations, and the adult prevalence rates are often between 20% and 80%. It has been revealed that natalizumab therapy is associated with changes in anti-JCPyV antibody indices over time [143]. Nevertheless, one could realize that there is little evidence for accurate anti-JCPyV antibody titers which has been mostly provided through some therapy-based studies. Therefore, more epidemiologic studies are needed to reveal accurate JCPyV presence amongst populaces, genders and ages, so as to obtain a more realistic control point. Some very old data revealed that anti-JCPyV titers increase with age, yet, undeniably, antibodies are an indirect way of defining an infection—even the risk for it. For example, an older man may be at lower risk for higher uni-infection anti-JCPyV antibody titers compared to a younger man, if they have the same serologic titers, simply due to the age-related antibody productivity and the potential future reinfections as compared with the elderly, and finally the older man would be differently treated (with regards to their medical condition) due to sole serologic titers without realistic evidence.

Furthermore, several studies have revealed anti-JCPyV serologic status inversion, and even if sudden positivity is more logical due to a new infection, the reverse case seems to be confounding in several ways, i.e., a false test result, the existence of cellular immunity even in the decomposition of some antibodies, etc. [102]. So, is it really accurate that some therapies are associated with changes in anti-JCPyV antibody indices over time, or do we need further precise evidence to define these assumptions? Serologic status is personal for all cases; for instance, cases with low overall IgG titers may have reasonably low anti-JCPyV antibody titers, and vice versa. Nevertheless, the golden standard nowadays seems to be the Polymerase Chain Reaction (PCR), and additionally, antigen tests contribute to the logical identification of infection, rather than defining the serologic response of each individual. Thus, it would be more accurate to test for JCPyV nucleic acid and even antigens, so as to obtain a realistic JCPyV status. Since this is the era of molecular diagnostics and testing assays, it is required that they be applied in neurosciences as well.

Moreover, it has been said that the primary infection occurs during childhood, with the virus to remain latent in the kidneys and lymphoid tissue. Thus, even if PCR detects genetic fragments that can be either active or inactive, antigen tests—where a high antigenic load may more precisely indicate an active virus—may be applied to detect viral motility inside a person via diverse sampling, and, really, a moving virus is more likely to relocate to the CNS. For example, sole urine specimens could indicate the initial infection, but future sampling should target other viral secondary tissues so as to detect a potential viral motility, thus a potential risk factor for PML; even a high blood JCPyV load may be somewhat practical.

Undeniably, nowadays, neurotoxins such as ethanol, pesticides, lead, radiation, fluoride, toluene and manganese are evident in daily life [144]; the term “toxic leukoencephalopathy” encompasses a wide range of conditions that can injure and cause structural modifications of the white matter, and the insults can be toxic, metabolic, secondary to immunosuppression or chemotherapy, infectious or environmental in origin [145]. One could argue that different toxins and different rates of exposure can trigger different patterns in toxic leukoencephalopathy. Yet, PML and toxic leukoencephalitis are supposed to have different lesion pattern, but could PML be an acute toxic encephalitis or a just form of it or an advanced state of it? PML has been vastly reported in cancer patients, but leukoencephalopathy is a demyelinating process that seems common after radiotherapy and some types of chemotherapy such as intravenous/intrathecal methotrexate, so could cancer-related PML be a direct side effect of cancer management? [146]. Indeed, a critical state-of-the-art review highlighted that diseases are different manifestations of systemic inflammation that occurs due to toxins, and various symptoms and signs can accompany it, from mild-to-moderate to severe and critical conditions, and including various organs and systems [129].

Literature data on the so-called JCPyV and its tumorigenic actions seem to be contradictory even in animals [15]. In humans, it has been discussed that the LT-ag of SV40 and JCPyV can bind various proteins such as transcription factors, DNA repair and replication enzymes, tumor suppressors, anti-apoptotic proteins, signaling proteins and other proteins involved in the architecture of the cytoskeleton and intracellular trafficking -p53 and retinoblastoma protein, [147]; however, on the other hand, according to Hodgkin’s lymphoma, it was stated in previous sections that LT-ag bind tumor suppressors, meaning that the supposed JCPyV can act as a tumor suppressor. Therefore, given the fact that JCPyV can act as a tumor suppressor, how can the virus trigger the so-called PML in cancer cases and aggravate their preexistent condition? Moreover, in critical immunosuppression cases, could the virus act as an oncogenic so as to help the host to produce more cells in order for it to survive and proliferate [148]? In this scenario, these supposed JCPyV sequences act against the immunomodulation drugs, so as to keep the host in its natural state, and this scenario seems somewhat logical considering that PML with JCPyV positivity is common in lymphoma patients and immunosuppressed individuals; indeed, it was discussed in previous sections that the virus is highly detected in human leukocytes! Therefore, one could argue that in reality immunomodulation is highly tumorigenic! Yet, the hypothesis that the supposed JCPyV can be tumorigenic seems peculiar since the first reported cases [1] already had leukemia! So, perhaps these viral sequences had tried to act as tumorigenic in order to work against possible cancer medications or a potential toxicity could trigger PML with a random concomitant viral positivity; indeed, the 1958 cases expressed PML after radiotherapy, a factor that can trigger leukoencephalopathy by itself! Nevertheless, some authors conclude that the so-called JCPyV might be involved in cancer progression rather than in tumorigenesis [149].

Furthermore, in tumors with MCPyV positivity, the viral DNA had a clonal integration pattern in the human cell genome, and as observed for other polyomaviruses, the full BKPyV genome or fragments containing some early genes may transform various cell types from divergent animals in cell culture systems, yet transformation by BKPyV seems inefficient [47]. Could JCPyV have transformed human cells too, or has it already done so and such PML diagnosis based on the molecular diagnosis of the virus is ambiguous due to that positivity because of a possible transformation? Bovine polyomavirus was detected in kidney cell cultures of the stump-tailed macaque but antibody presence was not reported; yet, these sequences exhibit homology to monkey genomic DNA sequences [150]. So, one could argue that it is a monkey genomic DNA from a destroyed cell due to inflammation rather than a virus! Given the fact that this virus shares homology with JCPyV, and that monkeys and humans also share genomic homologies, could this be possible even for JCPyV? In this case, this sequence that is believed to be a virus named JCPyV could possibly be a human genome sequence that naturally does not coexist with any symptomatology, but after potential inflammation and cell apoptosis/necrosis (due to toxins that already cause symptomatology) this could be released in the microenvironment and as a result it could be highly detected by molecular diagnostic methods. It is assumed that the so-called JCPyV is transmitted horizontally, but it seems to be passed most often from parent to child (vertical transmission), and established infections can persist in the host for extended periods of time [19].

Interestingly, a study showed that JCPyV-positive Africans had European mtDNAs, and given the fact that the supposed JCPyV is assumed to have European ancestry, the initial viral sequence may have been detected in European mitochondria [151]. Therefore, molecular diagnosis may not always be positive regardless of clinical manifestations giving rise to the supposed asymptomatics [152], but also, this scenario seems to bring us closer to the real answer about the existence of JCPyV! Since there may be a vertical transmission that can be asymptomatic, why are there no existent virions in the 1971 cell culture controls [2]? Moreover, regarding the lymphoma case of 1971, there is no positivity in serologic anti-JCPyV tests [2]. It was previously discussed that paraproteins in lymphoma can result in false serologic test results, so could this be a false negative one? However, PML is highly reported in cancer and especially in patients with lymphoma, so could that be a real false-positive due to paraproteins? Also, in the 1971 case report [2], a molecular identification was not made, and also the patient was seronegative for most known viruses—meaning even for the already known polyomaviruses—but how can this be possible? The so-called JCPyV shares homology with other polyomaviruses, thus even a possible false-positivity should be evident! It was discussed that BKPyV bears resemblance to JcpYV in terms of its wide sero-prevalence, thus a false-positive ought to be evident in this old case report!

Moreover, it has been discussed that individuals with type 0 blood may be more likely to express PML manifestations, and that the presence of JCPyV on B lymphocytes’ surfaces in cases with type 0 blood can promote the aggregation of lymphocytes and erythrocytes, resulting in cell clumping which becomes impacted in narrow cortical capillaries with low blood flow at the gray-white junction; yet, if the serum of a person that is infected with the virus is mixed with RBCs and the virus, RBCs will not agglutinate (hemagglutination inhibition), and antibodies that are present in the infected person’s serum neutralize the virus leading to a positive result! [33]. Indeed, bone marrow B lymphocytes and CD34+ hematopoietic precursor cells are prone to JCPyV infection and they seem presumed to be a viral reservoir involved in viral carriage to the brain, particularly when such cells are increased in peripheral blood—like during natalizumab treatment [32]. One could argue that in this way, the risk for blood cancer seems to be higher; actually, except from HIV and immunosuppression, PML seems more likely to occur in lymphoma cases.

JCPyV genome sequences have been detected in several other disorders [22]. Therefore, could this be a random false-positive considering vertical transmission? Differential diagnosis that was discussed in previous sections includes meningitis, and since JCPyV can be positive in fatal meningitis, then a vaccine against meningitis could be questioned; could this specific case be a false-positive? [153]. Then another question arises, regarding the reality of PML: is it really PML or another known disease with just a different phenotype? [154]. For instance, it is well known that in natalizumab-associated PML, CSF analysis is somewhat different from other non-natalizumab-associated PMLs; is it the serologic overload due to MS or is it the drug itself that affects such analysis and imaging features and the clinical phenotype as well? [155].

Given the fact that most people are positive (mostly due to vertical transmission), one could argue that in every neuroinflammatory condition—where there occurs cell apoptosis/necrosis and such genomic sequences are revealed into the microenvironment—there is a high risk for a molecular positivity, thus there arises a risk for a false-positive diagnosis of PML! In that case, all negatives are in reality false negatives, since most of us have such sequences in our genomes, thus most neurological diseases ought to be accompanied by JCPyV positivity! A study showed that there is a high rate for false negatives even for the assumed PML [104].

Up to now, the literature has revealed few reported PML cases with no preexistent immunosuppression, HIV, cancer, or autoimmune-supposed conditions that directly depict a direct lymphatic system dysfunction (spleen and bone marrow) and further kidney infiltration, thus it is obvious that it is the preexistent state that may worsen and lead even to PML manifestations rather than the supposed virus itself. Moreover, kidney disease is common in Hodgkin’s lymphoma, immunosuppression and HIV, since lymphatic drainage seems difficult, thus JCPyV detection may be sensitive due to kidney inflammation (destroyed cells thus leading to higher positivity in JCPyV). Indeed, it was discussed in previous sections that the so-called JCPyV exists mostly in bone marrow and spleen—in other words, the lymphatic system, where fluid body levels throughout the body are managed—and it is known that CSF convects via the cribriform plate into lymphatic vessels in the submucosa linked to the olfactory and respiratory epithelium [156]. Even in the first 1958 case reports on PML [1], it is evident that these cases were full of preexistent medical conditions and in one case the lymphatic dysfunction was obvious (spleen reported issues), so they presented excessive underlying systemic inflammation [129], thus undeniably PML manifestations were affected by all these factors. A study during the COVID-19 pandemic revealed that the incidence of various underlying pathologic disorders is high [157], therefore systemic inflammation is highly obvious [129], raising further questions about future PML risks and clinical manifestations.

Regarding PML pathology, it has been experimentally demonstrated that the supposed JCPyV can lead to non-apoptotic cell death in infected glial cells, and also, in the first PML case reports [1,2], there were no signs of apoptosis or necrosis but some bizarre cells had been noticed, but experimental conditions are far away from the in vivo conditions! Also, it has been discussed that the atypical astrocytes are critically pleomorphic and they can have one or more irregular nuclei with condensed chromatin and a clear nucleolus and also both nucleus and cytoplasm small inclusion bodies can be evident; lysis has not been detected, but some foamy macrophages have been noticed and are presumed to be phagocyte-released oligodendrocyte myelin and perivascular cuff of lymphocytes [103]. Therefore, even if JCPyV sequences are possibly included in the human genome, necrosis is more likely to occur, and given the fact that JCPyV sequences are integrated into the human genome, then exogenous toxins trigger necrosis. Such neurotoxins were previously discussed; hence, ostensibly, we speak about forms of toxic leukoencephalopathy and not really PML. In addition, one could argue that perhaps necrosis did not have time to take place or to be seen, or samples may have been received from relatively active lesions, compared to some others that had been inactive in prior times.

Apparently, there are several crucial areas where the supposed PML seems incomplete, yet the finding of both supposed Archetype and neurotropic strains in a different subpopulation of peripheral blood mononuclear cells suggests that the blood compartment might be the site of the assumed JCPyV conversion; given that in reality we speak about toxins, then the blood compartment is the initial site of toxin entry! One could argue that such neurotoxins target neurons but they do not reach them directly due to the BBB and oligodendrocytes, and that may be the reason these cells were identified to be harmed in the initial case reports. Nevertheless, is it a real neurotoxin or generally a toxin for the human body?

Could peripheral toxins enter the brain through lymph (leukocytes)? Could a poor peripheral lymphatic drainage lead to poor CSF drainage near lymph nodes resulting into lymphedema and the so-called mass effect in some PML lesions? [158]. It has been discussed that impaired functioning of the meningeal lymphatic vessels gives rise to the accelerated accumulation of toxins into the brain parenchyma, thus triggering various pathologies analogous to each toxin [159]. In this scenario, apoptosis/necrosis may occur in the astrocytes due to toxins and dysfunction (astrocytes control blood flow, transfer mitochondria to neurons, and supply the building blocks of neurotransmitters that fuel neuronal metabolism, thus they modulate the brain metabolism) [160] as well as some other glial cells derived from progenitor monocytes due to accumulated toxins in the BBB. Moreover, the near oligodendrocytes will be unprotected, thus demyelination during inflammation in the microenvironment is inevitable. So, since glial cells are unable to protect neurons, and given that toxins may be in the blood circulation in general, bone marrow may be forced to produce more leukocytes in order to manage toxins and further inflammation; therefore, the risk for blood cancer is evident! By this way, it seems that (neuro)toxins preexisted in lymphoma cases who manifested PML! Nevertheless, it is more likely for systemic inflammation to preexist in the body rather than beginning in the brain, since not only the gastroenteric system serves as an entry for possible toxins to the blood circulation and thus the whole body, but also the brain microenvironment—particularly the neurons—are strictly controlled and guarded by so many glial cells, apart from the common leukocytes that exist in the whole body, too. In this way, initially, the supposed PML may lead to cortical lesions, because they are linked to meningeal inflammatory infiltrates in these regions which are closer to meningeal lymphatics as well, whereas other lesions seem to be deeper in the brain parenchyma due to extensive inflammation and leukoencephalopathy [161]. MS cases already have demyelination, and the mechanism of action of natalizumab decreases lymphocytes’ entry into the brain, thus it is logical that there is a higher risk for such cases to manifest the so-called PML; in this way, the whole lymphatic drainage becomes poorer and the risk for malignancies becomes higher.

It is plain to see that a direct supposed PML pathology cannot be established, since molecular tests are not infallible, imaging features are not directly accurate in all cases, symptomatology is not always illustrative, and also, various different underlying conditions (such as AIDS and lymphoma) or/and medications (like chemotherapy and natalizumab) can result in different pathology, thus all previous discussion on its pathology may be just speculation, and of course, it was discussed in the symptomatology section that various factors affect clinical manifestations and symptomatology. Since various conditions can lead to divergent supposed PML phenotypes, various lesion types can trigger a wide range of signs and symptoms, and in this way, even lesions with no mass effect may be fatal for a case with significant preexisting systemic inflammation.

Finally, JCPyV seroconversion rates have been reported to be stable during the COVID-19 pandemic, [162], but some data indicate that HIV/AIDS prevalence may increase in the post-COVID-19 period [163,164,165,166,167]. Rare reports describe novel PML manifestations due to SARS-CoV-2 [168]. Even if the incidence of PML has increased dramatically after the onset of the AIDS pandemic, and taking into account the previous facts on the COVID-19 pandemic, PML may be more common either in the elderly population or with some new forms as well as clinical and imaging manifestations and laboratory analyses. Additionally, PML-IRIS may be more common in the post-pandemic era, and perhaps new forms of it may be reported, since immunomodulation strategies for various underlying medical conditions are constantly evolving.

## Figures and Tables

**Table 1:** The medical diagnosis of the so-called PML [9].

PML	Clinical Manifestations	Imaging Features	CSF PCR Test
Definite	+	+	+
Probable	+ −	− +	+ +
Possible	+	+	+
Negative	+ −	− +	− −

**Table 2:** The histopathological diagnosis of the so-called PML [9].

PML	Histopathologic Triad	JCPyV PCR	Immunohistochemistry/ Electron Microscopy
Definite	+	+	+
Probable	+		
Possible			+
Negative	−		−
